# Unexpected Dual Left Anterior Descending Artery as a Source of Percutaneous Coronary Revascularization Failure

**DOI:** 10.3389/fcvm.2016.00045

**Published:** 2016-11-16

**Authors:** Steven D. Hajdu, Salah D. Qanadli

**Affiliations:** ^1^Department of Radiology, Lausanne University Hospital, Lausanne, Switzerland

**Keywords:** DUAL, left anterior descending, recanalization, coronary, failure

## Abstract

Dual left anterior descending artery (LAD) is rare. A 61-year-old patient was referred because of angina and a positive stress test. Coronary angiography revealed a short LAD originating from the left main coronary artery. Computed tomography showed a long LAD originating above the right coronary artery sinus.

A 61-year-old man was referred for transcatheter coronary angiography because of stable angina and a positive bicycle stress test. His medical history was unremarkable besides mild hypercholesterolemia. The angiogram showed occlusion of the distal left anterior descending artery (LAD), with a severe ostial lesion of the second diagonal branch (Figure 1A). The left circumflex and the right coronary artery (Figure 1B) presented no significant lesion. The second diagonal branch was successfully dilated with a 2-mm balloon, while recanalization of the LAD occlusion was unsuccessful. In order to plan a second attempt, a preprocedural coronary CT angiography was performed to identify the course and caliber of the LAD and better characterize the occluded segment and assess side branch as well as bridging collaterals. Surprisingly, the CT scan showed a double LAD anomaly. The anomalous distal LAD originated from the right coronary sinus with a prevascular course easily appreciated in the 3D reconstructions (Figures 2A,B). This anomaly, classified as type IV by Spindola-Franco et al., is an uncommon and rare among congenital coronary artery anomalies and is usually asymptomatic (1). Because the vessel had a separated ostium just above the origin of the right coronary artery, it was not identified during selective coronary angiography.

**Figure 1:**
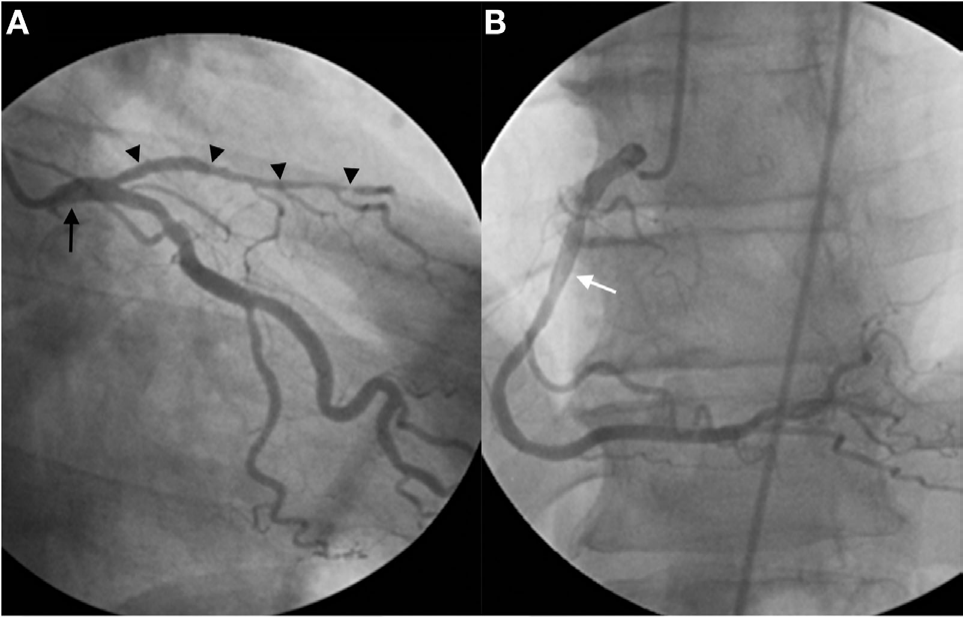
**Selective left coronary angiography shows a short LAD (arrowheads) originating from the left main coronary artery (arrow), giving rise to septal branches and diagonal artery branches (A)**. Selective right coronary angiography reveals a dominant RCA (white arrow) without any additional vascular anomalies **(B)**. Attempted recanalization of the distal portion of the short LAD was unsuccessful.

**Figure 2:**
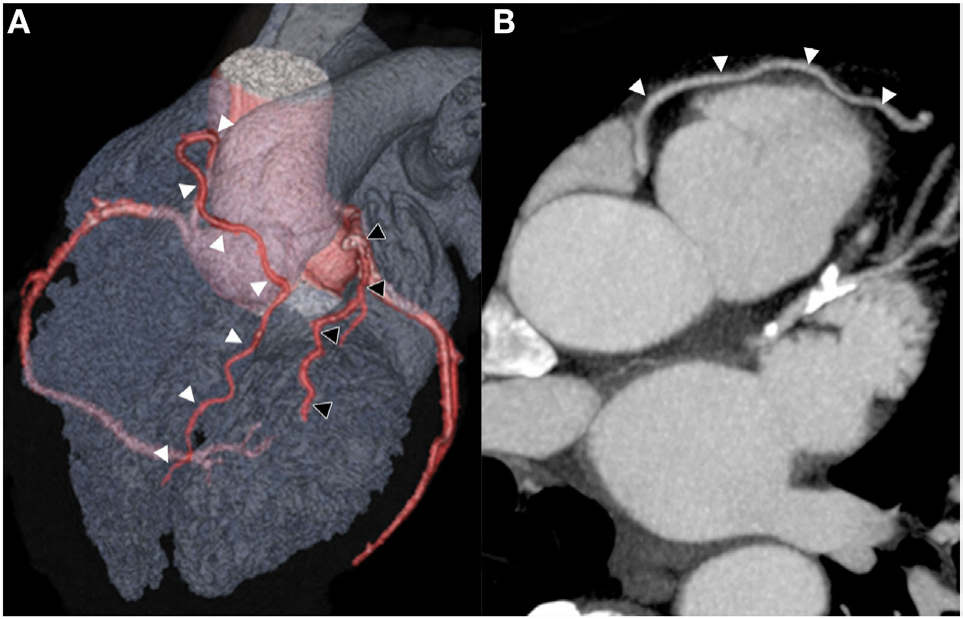
**An anterior projection of a three-dimensional volume-rendered computed tomography image confirms the short LAD (black arrowheads) originating from the left main coronary artery terminating its course in the interventricular sulcus**. Additionally, a long LAD (white arrowheads) is visualized originating above the right coronary artery sinus initially pointed cranially, then traveling anteriorly to the interventricular sulcus and finally descending toward the apex **(A)**. Axial computed tomography image demonstrates a long LAD epicardial course (arrowheads) as it passes anteriorly to the right ventricular outflow track, consistent with a Spindola-Franco class IV dual LAD **(B)**.

The presented case clearly demonstrates the potential role of CT to help understand failure of percutaneous coronary interventions as well as its potential role to plan optimally recanalization of coronary chronic total occlusions.

## Ethics Statement

This case report was exempted from any ethics committee verification due to its retrospective nature. The radiological images and case presentation were approved by the patient to be used for publication.

## Author Contributions

Both the authors contributed substantially and equally to the conception, analysis, and creation of this manuscript.

## Conflict of Interest Statement

The authors declare that the research was conducted in the absence of any commercial or financial relationships that could be construed as a potential conflict of interest.
